# Editorial: Computational Predictions, Dynamic Tracking, and Evolutionary Analysis of Antibiotic Resistance Through the Mining of Microbial Genomes and Metagenomic Data

**DOI:** 10.3389/fmicb.2022.880967

**Published:** 2022-04-04

**Authors:** Liang Wang, Alfred Chin Yen Tay, Jian Li, Qi Zhao

**Affiliations:** ^1^Department of Bioinformatics, School of Medical Informatics and Engineering, Xuzhou Medical University, Xuzhou, China; ^2^Jiangsu Key Laboratory of New Drug Research and Clinical Pharmacy, School of Pharmacy, Xuzhou Medical University, Xuzhou, China; ^3^Laboratory Medicine, Guangdong Provincial People's Hospital, Guangdong Academy of Medical Sciences, Guangzhou, China; ^4^The Marshall Centre for Infectious Diseases, Research and Training, University of Western Australia, Perth, WA, Australia; ^5^School of Public Health and Tropical Medicine, Tulane University, New Orleans, LA, United States; ^6^School of Computer Science and Software Engineering, University of Science and Technology Liaoning, Anshan, China

**Keywords:** antibiotic resistance, microbial genomes, metagenomic data, evolutionary analysis, computational predictions

Due to the continuous misuse of antibiotics globally, antibiotic resistant arises from antibiotic resistance genes (ARGs) that are now widely detectable from a variety of environmental water and soil resources and various microbial species such as *Escherichia coli* and *Klebsiella pneumoniae* (Von Wintersdorff et al., 2016). As a result, fast and efficient molecular tools are very important to aid the identification, determination, and profiling of antibiotic resistance in environmental samples. In addition, bioinformatics analysis of the microbial genomes and metagenomic data would greatly facilitate our understanding of the molecular mechanisms, environmental transmissions, and dynamic changes of antibiotic resistance (De Abreu et al., 2021). Recently, many advanced bioinformatic methods, including the use of metagenomic next-generation sequencing (Berglund et al., 2019; De Abreu et al., 2021), machine learning (Liu et al., 2020; Anahtar et al., 2021), and Raman spectroscopy (RS) (Tang et al., 2021; Liu et al., 2022), have been proposed to predict ARGs and their mode of action. However, with steady accumulation of massively sequenced data and continuous antibiotic resistance emergence, novel and effective methodologies and tools for ARG prediction and antibiotic resistance profiling analysis and visualization are constantly needed. In order to gain advantage in winning the antibiotic resistance battle, efficient and accurate computational tools are required to determine novel ARGs (Maryam et al., 2021). Under this Research Topic, we sought to highlight an exciting set of groundbreaking efforts proposed by frontline investigators, which mainly focused on implementing computational methodologies to get an in-depth understanding of microbial antibiotic resistance. Articles can be fitted into either of the four categories: (i) novel computational methods, (ii) development of computational tools, (iii) metagenomic data mining, and (iv) microbial genomic analysis. It is expected for some submissions to overlap between categories due to the comprehensive nature of the Research Topic.

In this special issue, majority of the submitted articles were focusing on the novel methods for the rapid and accurate analyses of antibiotic resistance. For example, Wei et al. compared the methods for selecting operational taxonomic units from 16s amplicon sequences. Such research could help biological researchers best select the reasonable clustering method for metagenomic analysis, and facilitate algorithm developers to design more efficient sequence clustering methods. For the discovery and identification of ARGs from fragmented metagenomic assemblies, Shafranskaya et al. presented a novel computational pipeline, termed GraphAMR, to improve read mapping technology. Moreover, Ivanova et al. established a novel bioinformatic pipeline to assist the High-throughput Chromosome Conformation Capture metagenomic analysis, including the identification of bacterial ARGs (or resistomes). Finally, there was a few studies that explored the antibiotic resistance issues from a non-genome-centric angle. For example, Wang et al. summarized recent applications of Raman spectroscopy technique in the antibiotic resistance profiling. They indicated that although there is still a gap between laboratory research and clinical applications for RS, rapid and reliable automatic measurement of the Raman spectra for antibiotic resistance profiling is promising, and eagerly and urgently in need. In another example, Ma et al. developed the Inductive Logistic Matrix Factorization, a novel drug-metabolite association prediction tool that can combine multiple-source interactions between drugs and metabolites and improve prediction performance of drug-metabolite associations, leading to potential applications in the development of novel antibiotics.

Apart from the novel computational methods, two powerful computational pipelines for general analysis of genomes and metagenomes were also presented in this special issue. In brief, Hierarchical Clustering with Kraken (HCK) and Abundance-Base Alternative Approach (ABAA), both developed by Mlaga et al., were designed to classify TS1 amplicons and to detect and filter non-specific amplicons in fungi metabarcoding sequencing datasets, respectively. These two novel pipelines, named HCK-ABAA, had improved the fungi community structures identification and stabilized methodology for metabarcoding analysis. In addition, Hua et al. developed a new web-based server to aid in annotation of AGRs, integrons, and transposable elements. This server could significantly accelerate the bioinformatics analysis of ARG-related sequences.

Two research papers were included in the Research Topic to directly investigate the computational analysis of metagenomic data from clinical perspectives. It is well-known that early, fast, and precise detection of antibiotic resistance is the key to an infection therapy. However, the determination of minimal inhibitory concentrations (MICs) in clinical settings via the conventional agar culturing methods can be very time consuming. To attack this problem, Tan et al., based on the analysis of metagenomic data via XGBoost algorithm and deep neural network (DNN) algorithm, combined single-nucleotide polymorphism (SNP) information and nucleotide k-mers count, and predicted MICs of meropenem against *Klebsiella pneumoniae*. This study significantly improved the ARG detection efficiency. In another study, Han et al. predicted several functional pathways via the computational analysis of fecal microflora composition of acute myocardial infarction (AMI) patients. This could enhance the comprehension of AMI pathogenesis.

Interestingly, a few articles had focused on the geographic distributions and identifications of multi-drug-resistant strains via computational analysis of bacterial genomes. For example, Chung et al. developed a mode-based web tool via Matrix-Assisted Laser Desorption Ionization-Time of Flight Mass Spectrometry to identify multi-drug resistant *Staphylococcus aureus*. In addition, Jiang et al. developed a SNP profiling technique based on whole genome sequencing to facilitate genomic population analyses of *Helicobacter pylori*. This approach holds the potential in understanding the global dissemination of antibiotic resistance genes.

In summary, the findings of the studies collected in this special issue could greatly help mankind in fighting antibiotic resistant in microbial pathogens, from a long-term perspective, and strengthen the faith in finally winning the invisible war all over the world. In addition, we want to thank all the authors who contribute their original work to our special issue and the reviewers for their valuable comments. We would like to express our sincere gratitude to the Specialty Chief Editor, Dr. Matthias Hess and Dr. George Tsiamis, and also the editorial office of Frontier in Microbiology, for their excellent support and providing us with this opportunity to hold this hot topic issue successfully.

## Author Contributions

LW drafted the manuscript. QZ revised the draft. AT and JL made substantial contributions to the work through in-depth discussion. All authors proposed the Research Topic theme, made a direct and intellectual contribution to the work, and approved the final version for publication.

## Funding

This study was supported by Foundation of Education Department of Liaoning Province (Grant No. LJKZ0280). LW is a bioinformatician and microbiologist who leads the Theoretical and Experimental Microbiology (TEM) Group https://tem-group.net/ at Xuzhou Medical University.

## Conflict of Interest

The authors declare that the research was conducted in the absence of any commercial or financial relationships that could be construed as a potential conflict of interest.

## Publisher's Note

All claims expressed in this article are solely those of the authors and do not necessarily represent those of their affiliated organizations, or those of the publisher, the editors and the reviewers. Any product that may be evaluated in this article, or claim that may be made by its manufacturer, is not guaranteed or endorsed by the publisher.
